# Cancer Immunotherapies: From Efficacy to Resistance Mechanisms – Not Only Checkpoint Matters

**DOI:** 10.3389/fimmu.2021.690112

**Published:** 2021-07-21

**Authors:** Shuyue Wang, Kun Xie, Tengfei Liu

**Affiliations:** ^1^ National Engineering Laboratory for Druggable Gene and Protein Screening, Northeast Normal University, Changchun, China; ^2^ German Cancer Research Center (DKFZ), Heidelberg University, Heidelberg, Germany; ^3^ Medical Faculty Mannheim, Heidelberg University, Heidelberg, Germany

**Keywords:** immunotherapy, checkpoint inhibitor, resistance, tertiary lymphoid structures, hyperprogression

## Abstract

The immunotherapeutic treatment of various cancers with an increasing number of immune checkpoint inhibitors (ICIs) has profoundly improved the clinical management of advanced diseases. However, just a fraction of patients clinically responds to and benefits from the mentioned therapies; a large proportion of patients do not respond or quickly become resistant, and hyper- and pseudoprogression occur in certain patient populations. Furthermore, no effective predictive factors have been clearly screened or defined. In this review, we discuss factors underlying the elucidation of potential immunotherapeutic resistance mechanisms and the identification of predictive factors for immunotherapeutic responses. Considering the heterogeneity of tumours and the complex immune microenvironment (composition of various immune cell subtypes, disease processes, and lines of treatment), checkpoint expression levels may not be the only factors underlying immunotherapy difficulty and resistance. Researchers should consider the tumour microenvironment (TME) landscape in greater depth from the aspect of not only immune cells but also the tumour histology, molecular subtype, clonal heterogeneity and evolution as well as micro-changes in the fine structural features of the tumour area, such as myeloid cell polarization, fibroblast clusters and tertiary lymphoid structure formation. A comprehensive analysis of the immune and molecular profiles of tumour lesions is needed to determine the potential predictive value of the immune landscape on immunotherapeutic responses, and precision medicine has become more important.

## Introduction

**Figure 1:**
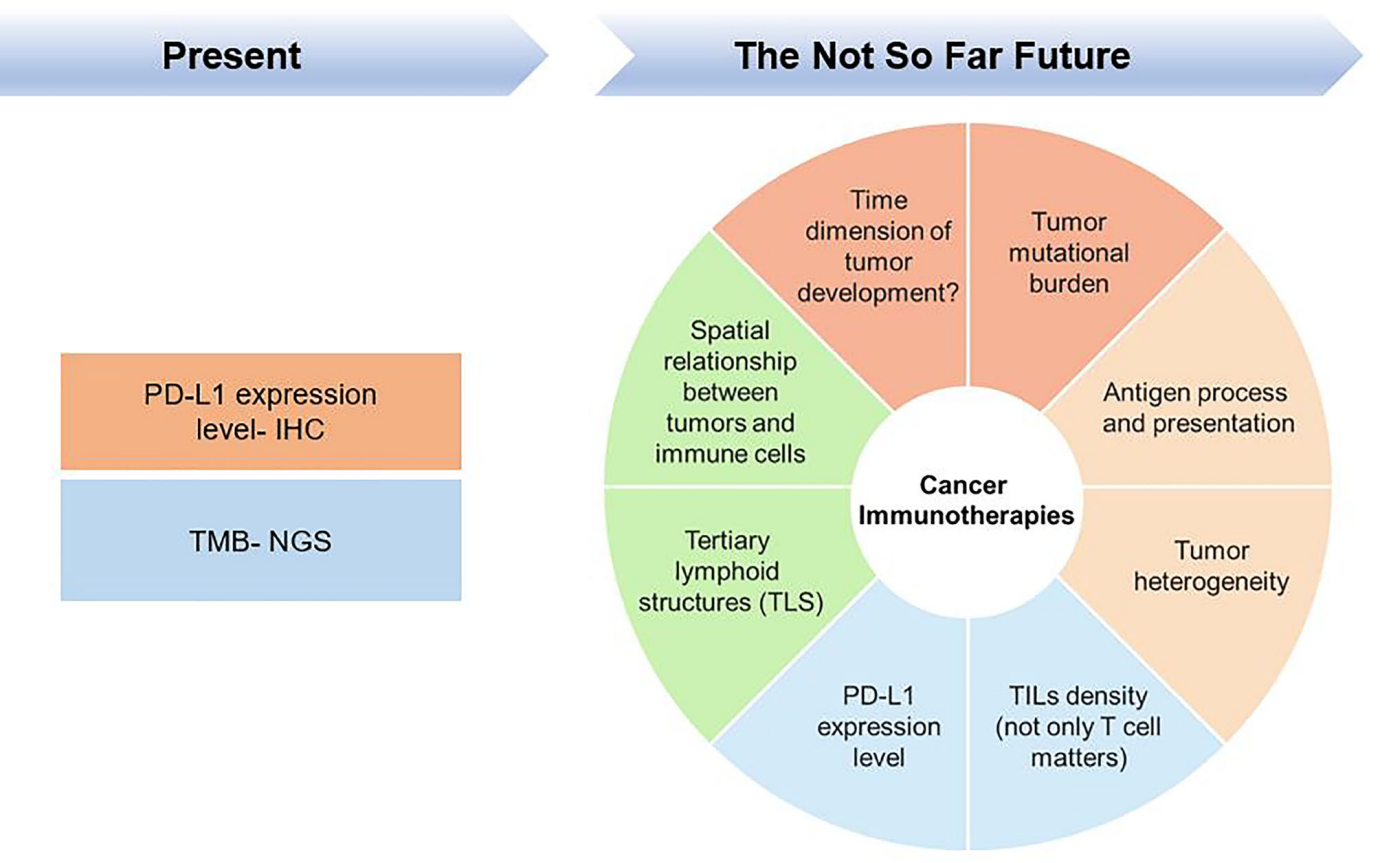
High-dimensional biomarkers are necessary for cancer Immunotherapies.

Over the past few years, remarkable results have been achieved with the availability of cancer checkpoint inhibitors (CPIs), which have revolutionized the oncology battlefield by making the host immune response a target for anticancer therapeutic intervention. However, a significant fraction of patients has no respond to CPI treatment; and moreover, a proportion of patients showed resistance to CPI, and some cancers may pseudo- or hyperprogress. Moreover, some patients are often heavily treated with different chemotherapy regimens prior to being treated with CPI, which increases the complexity of the TME. Taking anti-programmed cell death 1 (PD-1) monotherapy as an example, physicians or investigators have come to a clinical consensus that the PD-1 ligand 1 (PD-L1) expression level can serve as a criterion for treatment with CPIs, such as anti-PD-1 therapy. However, depended on the understanding of the tumour microenvironment (TME) and the tumour mutational burden (TMB), other related biomarkers are also used as criteria for CPI treatment. Immunotherapeutic research may be currently in the middle-to-late stage, and researchers have gradually realized that tumours and TMEs progress over time and thus must be reconsidered and evaluated from a continuous perspective rather than by simply using the expression of certain biomarkers at certain time points as single measures; the idea of high-dimensional biomarkers for tumour immunotherapy is depicted in **Figure 1** and will be addressed in this review.

Bearing this in mind, we firstly argued the intrinsic and extrinsic resistance mechanisms of immunotherapies and the advantages and shortcomings of using PD-L1 expression levels as biomarkers of PD-1 therapy and nonresponse to PD-1 treatment due to a limited understanding of PD-L1 cut-offs. And in turn we summarize the criteria for the immunotherapeutic treatment of “hot and cold tumours” from certain perspectives and try to determine the impact of immune cells on the TME in both temporal and spatial dimensions. Moreover, we also report some new findings on tertiary lymphoid structures (TLS) associated with hot/cold tumours to clarify that CD8 toxic T cell infiltration affects CPI treatment and that subtle structural features of the TME play an important immunotherapeutic role. We also discuss the problems and dilemmas of hyperprogression as an integral factor underlying immunotherapeutic resistance in cancer.

## PD-L1 as Biomarker in Immunotherapy: Applications and Shortcomings

Immunotherapies based on antibodies targeting the PD-1 and PD-L1 axis have profoundly changed the strategies for treated advanced tumours. Between 2015 and 2017, various ICIs were approved for the second- or first-line treatment of tumours with high PD-L1 expression (1).

Immunotherapies targeting PD-L1 and PD-1 are known to have substantial clinical impacts. First, anti-PD-1/PD-L1 inhibitors have broad pan-tumour potential and lead to better ORRs than former therapies in all patients. Second, patients treated with numerous PD-1 or PD-L1 inhibitors, such as nivolumab, pembrolizumab, durvalumab and atezolizumab, were shown to achieve sustained responses (2).

Despite these significant results, only a fraction of patients responds, and there is a strong need to define predictors to elucidate which patients are likely to have lasting clinical benefits. Most clinical trials have mainly investigated the predictive roles of PD-L1 expression in tumours and immune cells, as the main predictor, different companies were using different definitions, detailed list in **Table 1**.

**Table 1:** Strategies of different companies using PD-L1 expression level as a companion diagnosis (adapted from information released by College of American pathologist).

Agent	Atezolizumab (Genetech/Roche)		Nivolumab (BMS)	Pembrolizumab (Merck)	Durvalumab (AZ)
**Target**	PD-L1	PD-1		PD-1	PD-1
**PD-L1 IHC antibody**	Ventana SP142	Dako 28-8		Dako 22C3	Ventana SP263
**Cell types and scoring method**	NSCLC-TC/IC	NSCLC-TC		NSCLC-TC	NSCLC-TC
				UBC-TC/IC	
	UBC-IC				
**Cut-off definitions in NSCLC**	TC or IC≥1%	TC ≥1%		TC =1%-49%	TC ≥25%
	TC or IC≥5%	TC ≥5%		TC ≥50%	
	TC ≥50% or IC≥10%	TC ≥10%			
**Cut-off definitions in UBC**	IC≥10%; IC≥5%; IC≥1%	NA		≥1% TC or any stromal staining	NA

NA, Not Available.

Overexpression of PD-L1 in tumour cells leads to the escape of inhibitory pathways from host immune surveillance (3, 4), thus providing a scientific basis for the use of immune checkpoint inhibitors (ICIs) in cancer. The abnormally high expression of PD-L1 in TME may be due to the “primary” activation of various oncogenic signals and the “secondary” induction of inflammatory factors, such as IFN-γ (5, 6). In clinical practice, antibodies to PD-1 or PD-L1 rejuvenate “exhausted” T cells in TME, induce significant responses and sustained remissions, and have tolerable toxicity in patients with many types of cancer, such as lymphoma, melanoma, and mismatch repair-deficient tumours (6).

However, there is no general consensus, as not all PD-L1+ patients respond to immunotherapeutic treatment, and a proportion of PD-L1-negative patients respond. Patients treated with ipilimumab (an anti-CTLA-4 antibody) exhibited only a 20% sustained response rate for 5-10 years; pembrolizumab (anti-PD-1) also achieves an initial response rate of only 70-80%, which is reduced to 33% at the 3-year follow-up. Meanwhile, for the anti-PD-1 and anti-CTLA-4 combination clinical study, only 61% objective response was observed, and significant toxicity also exists at the same time (7).

In the face of such complex and variable outcomes, it is more important to consider the nature of immunotherapies and their relationship with the TME. In general, when considering immunotherapeutic efficacy, we may first consider whether the tumour is inflamed, which often indicates immune cell infiltration. In contrast, similar choices should be made regarding the use of biomarkers, such as PD-L1 expression in patients receiving anti-PD-1 or anti-PD-L1 therapy.

Patients may initially respond poorly to immunotherapy in the absence of tumour inflammation due to several reasons, such as immune compromise; the lack of antigen presentation, CD8 cell trafficking, T cell infiltration; and other issues in the TME. This also inevitably results in no recognizable antigens in the TME. Conversely, PD-L1 expression is presumably more relevant to the prediction of adaptive immune responses in cases of tumour inflammation. However, the following conditions affect the efficacy of CPIs for the treatment of inflamed tumours: 1) less T cell infiltration or T cell exclusion indicates a poor CPI response, and 2) more T cells in the tumour area indicates a more favourable CPI response (8). The concept of hot and cold tumours cannot be ignored and will be discussed in more detail later.

A few questions remain regarding the current situation and problems related to PD-L1 therapies, such as how to further enhance T cell function and how to convert noninflamed tumours into inflamed tumours. Why some patients respond, and others do not when using PD-L1 as a predictor also remains unanswered.

Researchers are realizing that tumours, as complex biological entities, cannot be measured by PD-L1 expression levels alone. The presence of TILs, the mutational load, and the likelihood of neoantigen expression in human cancers (different tumours have different likelihoods) influence clinical outcomes (**Figure 1**). Additionally, retrospectively evaluating the performance of PD-L1 prediction is not sufficient.

Therefore, the limitations of PD-L1 as biomarker of anti-PD1 therapy should be considered, and some confusion remains. Researchers should understand that different drugs, assay systems (such as clones, staining protocols, platforms and scoring methods), clinical decision points, tumour indicators and cut-offs will influence the conclusions.

And what is more, thinking in terms of different dimensions, PD-L1 as IO biomarker is dynamic and heterogeneous both spatially and temporally. PD-L1 expression can be also temporal and heterogeneous, this can make the situation even more complicated. This has forced us to rethink from the beginning of the mechanisms of immunotherapy resistance.

## Intrinsic and Extrinsic Mechanisms of Immunotherapy Resistance

Immunotherapeutic resistance is categorized as either primary resistance or acquired resistance. Primary resistance (also known as intrinsic resistance) is a clinical scenario that a cancer does not respond to an immunotherapeutic strategy. The incidence rates of various types of cancer can change drastically (9). The so-called hyperprogressive diseases (HPDs) were recently classified as being primarily resistant. Some studies suggested the main factors underlying HPD include intrinsic changes, such as murine double minute (MDM)2/4 gene amplification (10), alteration of chromosome 11 region 13 (such as CCND1, FGF3, FGF4, FGF19) (11), and epidermal growth factor receptor (EGFR) gene mutation (11). At the same time, there is a growing awareness that TME alterations, such as the polarization of specific types of macrophages (for example CD163+CD33+PD-L1+ macrophages), may also cause HPD (12).

Tumours that initially respond to immunotherapy effectively but either stop responding or grow over time are said to have acquired resistance (9). With the increasingly widespread use of ICIs, the number of patients with acquired resistance is gradually increasing. For example, approximately 1/3 of patients with advanced melanoma relapse after treatment (13, 14). In addition, the main mechanism of immunotherapy is in the activation of immune cells, while the resistance of adaptive immunity is another mechanism recently recognized by researchers, which is different from traditional chemotherapy, radiotherapy and targeted therapy. Tumours with adaptive immune resistance are identified by the immune system but escape death by altering themselves to suit to the immune aggression. Acquired resistance can be a primary form of resistance based on mixed responses to dynamic immune microenvironment modulation and the interaction between immune cells and cancer cells (9, 15). To date, plentiful mechanisms of immunotherapeutic resistance were well described and determined, and new mechanisms are continuing to be discovered.

### Intrinsic Factors Affecting Immunotherapy

#### Application and Shortcomings of TMB as a Biomarker

Extensive evidence suggests that the TMB is a predictive biomarker of immunotherapy response independent of PD-L1 (16, 17). The TMB is characterized as the mutations/Mb numbers in tumour cells and can be determined by next-generation sequencing platforms, including whole-exome sequencing (WES), whole-genome sequencing (WGS), and targeted panel sequencing (17). The rise of TMB can be driven by exogenous factors (tobacco carcinogen exposure, chronic viral infection, or ultraviolet light) or endogenous factors (such as impaired DNA repair) (17).

TMB is correlated with increased expression of tumour-specific antigens (TSAs) and tumour-associated antigens (TAAs) that can target reinvigorated T cells, whereas a high TMB tends to enhance tumour immunogenicity and ICI responses across tumour types (18–21). Several studies confirmed that increased mutational burdens were associated with higher response rates in patients with non-small-cell lung cancer (NSCLC) or melanoma treated with anti–PD-1 and anti-CTLA-4 antibodies, demonstrating the feasibility of using the TMB as a biomarker for patient selection. For example, researchers performed constitutional and somatic exome analyses of 77 nivolumab treated NSCLC patients and revealed that higher exonic nonsynonymous mutation and neoantigen levels were associated with a better outcome (22). In a study on patients previously treated for unresectable or metastatic solid tumours (KEYNOTE-158 study), a high TMB status (TMBH, ≥10 mut/Mb) was associated with a clinically meaningful improvement in the efficacy of the anti–PD-1 antibody pembrolizumab (23). On the basis of the KEYNOTE-158 study, the FDA approved pembrolizumab for the therapy of TMB high solid tumours. In the CheckMate 275 study, unresectable locally advanced or metastatic urothelial carcinoma patients which do not respond to a least one platinum-based regimen received nivolumab monotherapy. The results showed that 139 of 270 patients had an evaluable TMB, and TMB high (≥13 mut/Mb) was associated with a longer overall survival (OS), longer progression-free survival (PFS) and a higher objective response rate (ORR) for, and patients treated with nivolumab (24).

The TMB is associated with certain biomarkers, including MSI-H (high microsatellite instability), which results from dMMR (deficient DNA mismatch repair) and is detected in a subset of human cancers. Tumours with an MSI-H/dMMR status typically display high TMB levels, and MSI-H/dMMR is an established predictive biomarker of ICI efficacy (25–27).

As reported, MSI has the highest incidence in endometrial (~30%), gastric (~20%), and colorectal (~15%) cancers and likewise occurs in lower proportions in other different tumour types. About 20,000 stage IV dMMR tumours are diagnosed each year in the United States (20). A WES study revealed that dMMR tumours showed an average of 1782 mutations in which of 578 were predicted to induce neoantigens, which highlights the immunogenicity of these tumours (28). The MMR/MSI status was also identified as a predictive marker in PD-1 inhibitor (such as pembrolizumab) treated refractory dMMR patients. In one trial, 46 noncolorectal cancer (12 mixed tumour types) and 40 colorectal cancer (CRC) patients were enrolled, 53% patients achieved an objective radiographic observation, including 21% CR. The 2 years OS and PFS rates were 64% and 53%, respectively (20). Based on these impressive results, the FDA approved pembrolizumab as the first agent for the treatment of MSIH/dMMR cancers that progress after first-line treatment (29). Therefore, an MMR/MSI status in PD-1 refractory diseases can substantially affect the treatment approach. Elevated levels of PD-L1 were found in MSI-H tumours, particularly on tumour-infiltrating immune cells (30), thereby providing a putative biological basis for strong anti-PD-1 therapeutic responses in patients with dMMR/MSI-H tumours (31, 32).

However, the correlation of the TMB with ICI response is not consistent neither across nor within tumour types. As reported, renal cell cancers (RCCs), Merkel cell carcinoma (MCC), and mesothelioma have higher ICI response rates than predicted based on their TMBs, which is potentially due to the higher antigen qualities in these tumour types that result from a high number of indel mutations (in RCC), viral antigens (in MCC), and complex chromosomal rearrangements (in mesothelioma) (30, 33). Another recent study suggested that a particular subset of patients with prostate cancer can benefit from immune checkpoint inhibitor despite a low TMB. Moreover, the study also suggested that IFN-γ response gene signatures and CD8+ T cells infiltration level may improve patient selection for ICI treatment, particularly for cancers with low TMBs (34). Most recent findings show that not all solid tumours with TMB-H are sensitive to immune checkpoint inhibitors: Type I cancer types (endometrial cancer, microsatellite stable colon cancer, metastatic melanoma, and other cancers) were significantly associated with TMB in terms of objective remission rates and overall survival, respectively, and Type II cancer types (such as renal clear cell carcinoma, metastatic squamous lung cancer) were not. This may return researchers’ obsession with single-factor biomarkers back to a deeper understanding of biology itself rather than the formalities of large numbers of retrospective studies (35). Thus, to better understand and predict patient outcomes and refine treatment strategies, it is imperative to identify additional genomic factors that influence response.

#### Loss of Antigen Processing and Presentation Can Affect ICI Therapy

Antigen or neoantigen processes and presentation are essential for the T cell recognition of tumour cells and engagement of the T cell receptor (TCR). Neoantigens are newly generated antigens following somatic tumour mutations which confer the immunogenicity of tumour and are necessary for the effectiveness of PD‐1 and PD‐L1 monoclonal antibodies (36). In addition, proteins involved in antigen processing, transport and presentation, such as beta 2 microglobulin (β2M), human leukocyte antigen (HLA) molecules, large multifunctional protease (LMP), and transporter-associated with antigen processing (TAP) are important for tumour antigen processing and presentation, and genetic modifications of these proteins can lead to ICI resistance (37). Some previous studies revealed low HLA expression level in melanoma, lung cancer and breast cancer and discovered it to be associated with primary resistance to ICIs and poor clinical outcomes (38–41). Earlier research showed mutations in the β2M gene in CRC and revealed that β2M mutations were remarkably related to the MSI phenotype and had a lower prevalence in microsatellite stable (MSS) tumours (42, 43). Consistent with this finding, anti-PD-1 monoclonal antibody resistance was reported in MSI status carrying β2M mutations CRC patients (20). The same phenomenon was also observed in melanoma patients who acquired resistance to PD-1 blockade due to a homozygous truncating mutation in β2M, which prohibited the major histocompatibility complex (MHC) class I surface expression level (13). Furthermore, RNA sequencing and flow cytometry analyses of melanoma patients showed that downregulation of MHC class I molecules is a hallmark of PD-1 inhibitor resistance that is associated with TGF-β, upregulation of SNAI1, CAF (cancer-associated fibroblast) related signatures and the MITF-low/AXL-high melanoma phenotype. Anti-PD-1 combo with other drugs that aim the TGF-β signalling pathway to reverse melanoma dedifferentiation may select as effective strategies of cancer therapies in the future (44).

While loss of antigen presentation has been found to be associated with ICI resistance, it may also be a component of metastatic heterogeneity with clonal evolution (45). Downregulation or total loss of HLA expression on tumour cells is a known mechanism of cancer immune evasion and may contribute to ICI resistance (46). Additionally, HLA loss of heterozygosity (LOH), or disruption of neoantigen presentation ability, is considered to be a pattern of immune escape (47–49). HLA LOH was shown to occur more frequently than HLA or β2M mutations in patients with early-stage NSCLC (48). In addition, alterations in the HLA phenotype are often induced by mutations in the gene encoding the HLA class I heavy chain located on chromosome 6p21 or in the gene encoding the light chain of the HLA complex, β2M, located on chromosome 15q21. LOH-15q21 and LOH-6p21 frequently overlap in CRC, bladder cancer and melanoma, and the high incidence of LOH-15q21 in some malignancies, especially the overlap of LOH-15q21 with LOH-6p21, may have a significant impact on tumour immunogenicity and the efficiency of cancer immunotherapy (50). Thus, HLA LOH and intratumour heterogeneity (ITH) strongly impact tumour immunogenicity and the efficiency of cancer immunotherapies (51). An increase in HLA LOH is usually accompanied by an increase in the number of sub clonal mutations, thereby increasing the ITH, which is related with poor immunotherapy response (52, 53). Conversely, for melanoma patients, maximal heterozygosity of HLA-I loci improved the OS of patients treated with ICB compared with people who are homozygous for at least one HLA locus (49).

#### Lack of Viral Antigens and Cancer/Testis Antigens Affects Immune Response

Five already known oncogenic viruses, human papillomavirus (HPV), Epstein-Barr virus (EBV), human T cell leukaemia virus (HTLV 1), human herpesvirus 8, and Merkel cell polyoma virus, are relevant with around 15% of malignant tumours (54). For example, approximately 40% of non-Hodgkin lymphoma (NHL) cases and Hodgkin (HL)in immunocompetent hosts and with 95% of nasopharyngeal carcinoma (NPC) patients are associated with EBV (55). Virus-derived antigens are widely accepted to be important targets for T cell immune responses because it is usually immunogenic and highly expressed in tumour cells. However, tumour cells in patients infected with these viruses express a limited array of antigens, such as LMP1, LMP2, EBNA1 etc., which also have low immunogenicity. Bollard et al. (56) made autologous virus-specific T cells (VSTs) containing T cell clones that recognized LMP1 and LMP2 antigens and then found that 28 of 29 patients with relapsed EBV+ HL or NHL injected with the above cells as an adjuvant therapy kept in remission, while 13 out of 21 patients with resistant or relapsed disease with clinical responses (57, 58). Several groups have also reported responses in nasopharyngeal carcinoma (NPC) (57, 58), and one study reported that NPC patients treated with standard chemotherapy in combination with rapidly generated EBV-specific T cells (EBVSTs) had a 71.4% response rate and a significantly higher survival rate than the historical controls receiving chemotherapy alone (59).

Furthermore, tumour immunotherapies mainly depend on the tumour-associated antigens expression and the responses of T cells to tumour antigens. Cancer/testis antigens (CTAs) are encoded by 276 genes, such as LAGE-1, MAGE-A, SCP-1, NY-ESO-1 and TTK, from more than 70 gene families (60). CTAs are frequently expressed in different types of cancers but have restricted expression patterns in normal tissues. The frequency of CTA expression is highly variable depending on the tumour type (61). Moreover, because several CTAs are immunogenic and represent potential defined targets for antigen-based vaccinations and antigen-directed immunotherapies, they are considered to be potent cancer vaccine targets for clinical trials (62). CTAs can be used as cancer biomarkers for the diagnosis and selection of cancer treatment strategies. In oesophageal cancer, NY-ESO-1, MAGE-A, TTK and LAGE-1 are highly expressed and induce specific cytotoxic T lymphocytes (CTLs) to exert specific killing effects on tumour cells, and it was demonstrated by several clinical trials that immunotherapies are effective for oesophageal cancer (63).

However, the absence of characterization of genes associated immune responses in cancer cells has hindered further development of indicators for selecting and optimizing immunotherapy.

#### Increased Tumour Heterogeneity Affects ICI

Increased tumour heterogeneity (ITH) describes the variability between cancer cells within a single tumour. Cancer originates from a tumour cell clone that acquires the ability to proliferate uncontrollably while evading detection and clearance by the immune system. As the cancer progresses, genomic instability leads to the development of tumour cell subclones that acquire various genomic alterations (64) in which alterations are selective proliferation or survival advantages. One instance is the genomic alteration of genes essential for T-cell immune elimination and recognition (65). Among millions of tumour cells, genomic alteration induces dominant subclones that coexist and populate the entire tumour. The emergence of new technologies such as multi-region and single cell sequencing has provided increasing evidence of tumour cell subclones that harbour distinct genomic alterations (66, 67).

Clonal expansion occurs due to genomic alterations, individual mutations and changes in gene expression between tumour compartments. In patients with metastatic tumours, heterogeneity between the primary tumour and metastases is thought to underlie the complexity of the cancer (68). For example, tumour heterogeneity is considered to be one of the characteristics of uroepithelial carcinoma, which may be associated with a high mutational burden that changes the polarization state of the cells with each cell division and proliferation over time (69, 70). Treatments targeting individual genomic targets may result in the expansion of nonresponsive clones, whereas less targeted treatments (e.g., chemotherapy and immunotherapy) may substantially alter the clonal and transcriptional subtypes of individual tumours (71). Next-Generation Sequencing (NGS) technology is currently used to identify and characterize heterogeneity in urothelial carcinoma at the transcriptomic and genomic levels and offers the possibility to correlate tumour pathological alterations with clinical outcomes, but the heterogeneity of the tumours themselves remains a considerable obstacle to the development of new drugs or the selection of therapeutic strategies for patients with urothelial carcinoma (72–74).

#### Other Innate Anti-PD-1 Resistance Signature

PD-1/PD-L1 blockade induced primary and acquired resistance suggests other therapeutic mechanisms and biomarker possibilities for tumor patients. Hugo et al. analysed pre-treatment melanoma biopsies of the somatic mutagenomes and transcriptomes to recognize factors potentially influencing resistance to anti-PD-1 therapy or innate sensitivity (75). While a high mutational load was related with improved survival in both responding and nonresponding patients, responding tumours had more BRCA2 mutations. Thus, tumours with innate resistance also showed transcriptional signatures (called innate anti-PD-1 resistance, IPRES) that may play roles in the simultaneous upregulation of genes regulating mesenchymal transition, cell adhesion, extracellular matrix (ECM) remodelling, angiogenesis, and wound healing. Notably, MAPK-targeted therapy (MAPKi) induced a similar signal in melanoma, indicating that a nongenomic form of MAPKi resistance mediates cross-resistance to anti-PD-1 therapy (75). Validation of IPRES in other independent tumour cohorts was used to define a transcriptome subset across different advanced cancer types. These results indicate that impairing the biological process of IPRES may lead to improved anti-PD-1 responses in patients with melanoma and with other types of cancer. Consistent with this study, a study investigated DNA damage repair (DDR) pathway mutations in patients with CRC treated with ICIs and found that the incidence rates ATM and BRCA2 mutations were significantly higher than those of other genes. DDR mutations may function as biomarkers for patients with CRC treated with ICIs (76).

### Extrinsic Mechanism: Effects of the TME

#### TIL Density

In addition to PD-1/PD-L1, various studies have showed that tumour-infiltrating lymphocytes (TILs) in and around neoplastic cells reflect host immunity in a range of cancers, such as breast cancer (77), gastric cancer (78), and NSCLC (79), and that the density of TILs (mainly T cells and NK cells) is associated with clinical prognosis (77–81). The presence of immunosuppressive tumour stroma, especially in some solid tumours, hinders T cell infiltration, thereby limiting ICI efficacy (82). The baseline TIL status could also serve as an immunotherapeutic biomarker. For example, the clinical responses of PD1 or CTLA4 treated melanoma patients were associated with the intertumoural CD8+ T cell density (83, 84). T cell inflammation in the TME has also been associated with the clinical benefit of patients with advanced melanoma treated with immunotherapies, such as an anti–CTLA-4 mAb and high-dose IL-2 (85, 86).

Ishigami et al. (87) showed that patients with gastric cancer which had high levels of NK cell infiltration showed a better prognosis than those with low levels. Likewise, measuring the infiltration density of CD57+ NK cells and CD68+ macrophages in cancer component was shown to be a rapid, affordable, and proven useful method for predicting survival in patients with stage II+III CRC (88). In patients with stage II+III oesophageal cancer, the infiltrating NK cells density in the tumour stroma was significantly correlated with junctional status. In addition, the density of infiltrating NK cells in tumour nests and the density of infiltrating macrophages in both tumour nests and tumour stroma were remarkably correlated with patient prognosis after surgery (89).

#### Macrophages, MDSCs and Fibroblasts

The TME is responsible for the coexistence of immune cells and tumour cells, and the spatial distribution among cells, as well as the degree of cytotoxic T cell infiltration in the tumour nest, affects the efficacies of immunotherapies such as PD-1 to some extent. Both tumour heterogeneity and minute structural differences are the main factors underlying T cell infiltration. Thus, investigators have proposed the concept of hot and cold tumours, which will be reviewed in subsequent chapters. However, it should be noted that the concept of cold and hot tumours may not be applicable to only a single cell type (T cells), as the infiltration of myeloid cells (macrophages) and fibroblasts may also inhibit or promote tumour development. The complexity of this process increases over time and may alter the balance between tumour promotion and suppression depending on the degree of macrophage polarization.

It was observed that the infiltration of immunosuppressive cells in the TME, such as T regulatory cells (Tregs), myeloid-derived suppressor cells (MDSCs), M2 tumour-associated macrophages (TAMs) is always associated with immunosuppression as well as by the release of IL-10, TGF-β like immunosuppressive cytokines and other chemokines (90). The immunomodulatory effects of TAMs (91), MDSCs (92) and CAFs (93) can also enhance the immunosuppressive ability of the TME. In turn, the immunosuppressive cells can promote angiogenesis, which creates a vicious pattern of destruction by immune activation (76, 94).

TAMs are important microenvironment components of solid tumours that differentiate along the spectrum of M1 tumour-killing macrophages to M2 tumour-promoting macrophages (95). TAMs express chemokines such as CXCL8, CXCL10, CCL17 and CCL22, in addition to the immune checkpoint PD-L1, which attracts Tregs to tumour sites and downregulates immune responses (96, 97). In tumours, lactate in the TME also drives the polarization of macrophages into the immunosuppressive M2 phenotype (98). For instance, M2 macrophages are the predominant phenotype in oral squamous cell carcinoma (OSCC) and exhibit higher metastatic levels in tumour deposits (99). Moreover, cancer patients higher M2 macrophages rate have worse outcomes than those with lower rate, suggesting that immunosuppression induced by M2 macrophage may contribute to tumour progression and escape (99).

MDSCs also play an immunosuppressive role in cancer (100). As reported, MDSCs are recruited to the TME by various cytokines, including GM-CSF, CXCL8, MCP-1, CXCL1 and MCSF-1 (101–103). In head and neck squamous cell carcinoma (HNSCC) patients, MDSC levels are elevated in peripheral blood and tumours and supress the immune response *via* several mechanisms (104, 105). In the hypoxic TME, PD-L1 upregulated the numbers of MDSCs and other kinds of immune cells by HIF-1α to inhibit T cell activation (106). Furthermore, MDSCs also present peptides to T cells, leading to T cell surface molecules nitration and TCR dysfunction and which will lead to the antigen-specific T cell tolerance. In an *in vitro* assay, polymorphonuclear neutrophil (PMN)-MDSCs reduced around 75% T cell proliferation and around 80% IFN-γ release. Thus, the high frequency of PMN-MDSCs was closely associated with a poor OS, and CD11b+/CD16+ PMN-MDSCs subpopulation was most closely related with poor survival of HNSCC patients (104).

CAFs have been extensively shown to contribute to tumor heterogeneity, and that intratumoural gland types provide tissue heterogeneity that is correlated with clinical outcomes. For example, the stromal microenvironment shapes the intratumoural structure of pancreatic cancer, which may be correlated with ICI resistance, and CAFs may also directly contribute to the so-called tumour desert and exclusion conditions (107). The expression of genes related to CAFs was also found to be associated with T cell infiltration and resistance to nivolumab treatment (108). CAFs may affect ICI resistance *via* complex secretomic, matrisomic, surfaceomic and metabolomic mechanisms. First, the matrix fibre density organized by CAFs strongly influences the localization and migration of T cells (109). A study reported that the CAF-associated secretome directly and indirectly impairs antitumour immunity (110). The investigators also observed that inhibition of TGF-β expression, depletion of FAP-expressing cells and inhibition of CXCR4 in combination with CPI treatment could potentially inhibit ICI resistance in a mouse model (111).

However, some facets remain unknown, such as the key factors underlying the accumulation of suppressive CAFs in the TME, whether CAF subsets with distinct phenotypes and functions are derived from different cellular sources or different cellular states, and the level at which CAF-mediated CPI resistance can be targeted in the clinic.

#### Hypoxia and Gut Microbiota

Hypoxia is one of the main hallmarks of the TME. Cell proliferation is uncontrollable in hypoxic tumour environments, which eventually leads to vascular growth and to the limitation of oxygen and nutrients. Most solid tumours undergo rapid progression and aberrant angiogenesis (112, 113). In particular, hypoxia is associated with T cell-suppressor compounds secretion, such as adenosine and galectin-1 (114–116). Adenosine triggers the accumulation of intracellular cAMP which associate with immunosuppressive whereas galectin-1 is involved in the whole process of cell adhesion, invasion, and angiogenesis and is correlated with HNSCC patient’s survival rate (114, 116). TAMs preferentially accumulate in hypoxic tumours regions, and hypoxia plays a crucial role in TAM infiltration into the TME. In addition, TAMs in hypoxic tumour microenvironments are known to mediate resistance to multiple anticancer therapies and promote cancer recurrence (117).

Moreover, there is increasing evidence that the gut microbiota plays an important role in the immune response and cancer treatment. Zitvogel et al. hypothesized that the microbiota contributes to antitumour immune surveillance *via* the cross-reactivity of microbiota and tumour antigens, production of bacterial metabolites that may play a functional role in systemic regulation, as well as in the stimulation of pattern recognition receptors (PRRs) (118). Activated PRRs (expressed mainly by innate immune effectors) which can dictate the propensity to inflammation and immune stimulation or, the propensity to immunosuppressive responses (118). Several studies have confirmed that mouse models with different gut microbial compositions have significantly different treatment responses (119, 120). For example, it was reported by Sivan et al. that genetically identical mice from two different facilities with different commensal microbes exhibited differential tumour growth and immunotherapeutic responses, while cohabitation flattened these differences (120). Many of these hypotheses have been verified in patients with different cancers, such as melanoma, NSCLC, RCC and urothelial carcinoma, treated with immunotherapies (121–124). Frankel et al. reported in melanoma patients receiving immunotherapy, the metagenomic and metabolomic profiles of the human gut microbiota indicating that ICI responders were more enriched with *Bacteroidescaccae* (125). Broadly diverse microbiota compositions appear to be more common in patients who benefit more from treatment, and a large microbiota diversity is directly related to higher numbers of T cells in the blood and TME (121–123). There are early phase 1 studies designed to improve response among patients with anti-PD-1 resistant/refractory digestive cancers based on gut microbiota interventions in which investigators extracted the gut microbiota of healthy participants whose gut was similar to that of those with anti-PD-1 responsive digestive cancers to product FMT capsules and re-challenge anti-PD-1 immunotherapy in combination with FMT in cancer patients who had failed anti-PD-1 therapy (126). Given these findings, an increasing number of clinical trials have been carried out to further investigate the influence of the gut microbiome on immunotherapy (127), and how concomitant drug use alters the gut microbiota and ultimately the response to ICIs remains an area of interest.

## Turn Cold Tumor Into Hot

Cancer immunotherapy using ICI has revolutionized the treatment and physician’s perspective of advanced cancer. However, response rates to immunotherapy are still comparatively low in the major resistant cases. One main factor associated with initial CPI resistance is the lack of tumour T-cell infiltration, the so-called “non-inflammatory” or “cold tumour” feature. The lack of T cells in tumours may be due to lack of tumour antigens, lack of antigen presenting cells (APCs), lack of priming/activation of T cells, and impaired transport of T cells to the tumour bed.

As previously mentioned in the section on the extrinsic mechanism of ICI resistance, the formation of “hot” and “cold” tumours is complex and influenced by multiple factors, such as the chemokine distribution in the tumour nest, TLSs, B cell signature, and CAF-associated protein secretion and structures. Furthermore, defective recruitment of APCs or lack of T cell activation or co-stimulation after antigen presentation can be an influencing factor.

“Cold tumours” are usually defined as a lower infiltration rate of effector T cells in TME, a low mutational load, and a low neoantigen burden and are often characterized by an immunosuppressive TME (128). Several approaches have been utilized to activate cold tumours to some extent.

Many attempts have been used to try to turn cold tumours into hot ones, such as the intervention of various small molecule drugs, antibody drugs, combo therapies and even oncolytic viruses. We have listed a few interesting cases for reference. For example: Demonstrated in mouse breast, pancreatic and glioblastoma tumour models that anti-PDL1, anti-VEGFR2, and anti-LTβ receptor (LTβR) therapies were showed to induce high endothelial venules (HEVs) and to enhance cytolytic of TME, which leading to the destruction of tumours and transforming immune-cold glioblastomas into immune-rich ones (129). The genetic or pharmacological inhibition of Vps34 kinase activity using SB02024 or SAR405 (Vps34i) decreased tumour growth and improved mouse survival in multiple tumour models (melanoma and CRC) by inducing the infiltration of CD8+, CD4+ T effector cells and NK cells (130). Such infiltration resulted in the establishment of a T cell-inflamed TME, characterized by upregulation of the proinflammatory chemokines and cytokines CCL5, CXCL10, and IFN. Vps34i treatment induced the expression of STAT1 and IRF7, which are involved in the upregulation of CCL5 and CXCL10. Combination with Vps34i improved the therapeutic benefit of anti–PD-L1/PD-1 therapy in mice with melanoma and CRC and prolonged their survival. It revealed that targeting Vps34 converted cold tumours into hot inflamed tumours, thereby enhancing the anti–PD-L1/PD-1 blockade efficacy (130).

The TME includes a complex network of chemokine or cytokines which may affect cell trafficking to the tumour nest. Adhesion molecules also involve into the recruit effector T cells to the TME and to specific regions within the tumour. For example, CX3CL1 attracted majority of Th1 cells and effector-activated cytotoxic T cells, but CXCL9 and CXCL10 recruited more memory CD45RO T cells (131). In addition, chemokines can provoke the influx of immature DCs (iDCs) into the tumour bed (132). The absence of those chemokines and the consequential reduction in iDC influx into the tumour bed may underlie the reduced migration and activation of T cells at the tumour interface (133).

## Tertiary Lymphoid Structures

### Tumour Infiltrating B Cells and Tertiary Lymphoid Structures

New evidence indicates that tumour-infiltrating B cells have also been reported to play an essential role in the clinical outcome of cancer patients receiving anti-PD-1 therapy. A higher rate of melanoma-infiltrating B cells with a plasma cell phenotype prior to treatment was correlated with longer survival in patients treated with anti-PD-1 (134). It was reported by Petitprez et al. that sarcoma immune class E, featuring TLS-containing T cells, follicular dendritic cells (DCs), and dense B cells, was associated with better response rates and survival to anti-PD-1 therapy (135). In addition, higher densities of tumour-infiltrating B cells and TLSs were found in a group of melanoma patients receiving neoadjuvant therapy with anti-CTLA-4/anti-PD-1 antibodies in combination (136). Thus, both B cells and TLSs (lymphoid structures at the tumour front) may play important roles during ICI treatment.

TLSs, the ectopic lymphoid structures, are ectopic lymphoid organs that develop in nonlymphoid tissues at sites of chronic inflammation and have been identified in several types of cancer (137–139). Well-developed TLSs contain B cell zones with actively replicating B cell germinal centres (GCs) surrounded by a T cell region (140). HEVs and clusters of DC-lamp+ mature DCs are interspersed throughout TLSs (140). Similar in architecture to secondary lymphoid organs (SLOs), TLSs can arise in pathological conditions, including autoimmune diseases, pathogen infection, allograft rejection and cancer (141, 142). The occurrence, differentiation and localization of TLSs reportedly play important roles in the tumour immune environment and determine clinical outcomes (140–144).

### Prognostic Value of TLSs in Immunotherapy

Tumour-associated TLSs are often associated with good prognosis in the majorities of cancer types, including breast cancer, CRC, lung cancer and melanoma, demonstrating capacity to induce a systemic and long-lasting antitumour response (145, 146). However, TLSs and chronic intratumoural inflammation have also been associated with a tolerogenic tumour environment, which indicates that TLSs might increase cancer aggressiveness (147, 148)

In a retrospective study of NSCLC patients, researchers demonstrated that TLSs, referred to as tumour-induced BALT, were correlated with increased OS, disease-specific survival, and disease-free survival (DFS) (149). B-cell organizing into TLSs shows characteristics of a sustained humoral immune response, and high follicular B-cell density is correlated with longer survival in patients with NSCLC. the prognostic value is strongly enhanced by the combination of follicular B-cell and mature DC density in TLSs. Low densities of both follicular B cells and mature DCs may use to identify high-risk patients with poor survival (150).

For CRC patients, CD3+ TLSs are prognostic biomarkers in patients with both primary and metastatic CRC (151). T cell-enriched TLSs are associated with the immune component found in low-risk CRC, and immune events are enhanced by TLSs in local TME (152, 153). The TLS frequency is correlated with immune cell infiltration, which helps to improve the prognosis of patients with stage II CRC (154).

To present, several newer investigations have proven that cancer-associated TLSs have immunosuppressive, pro-tumorigenic effects. Indeed, the link between tumour-associated TLSs and patient outcomes seems to be dependent on many factors, including the type of TLSs, cancer type and disease stage.

### Efficacy or TLS Resistance in Immunotherapy

Increasingly evident suggest that a successful antitumour immune response requires the presence, activation and synergistic stimulation of all lymphatic components of the immune system, including CD8+ T cells, CD4+ T cells, B cells and innate lymphocytes within the TME. This is particularly reflected in the discovery of TLSs, which represent well organized clusters of TILs and elicit advanced immune responses (145). Assessing the impact of TLSs on treatment responses and their modulation by therapies has become necessary. Analyses of TLS densities as well as their location near or at a distance from the tumour nests, the composition and maturation rate, their effect on the clonality of T and B cell receptors within the tumour, and the production of antibodies by plasma cells educated by TLSs will likely be key in predicting therapeutic response and assessing therapeutic efficacy (155).

A high proportion of desmoplastic melanomas have been reported to exhibit formation of TLSs, and patients also have a high response to PD-1 blockade (156, 157). Two independent studies on human NSCLC reported that the presence of TLSs in lesions regressing after neoadjuvant anti-PD-1 therapy (158) or chemotherapy was correlated with longer DFS and OS (159). In contrast, caution should be exercised in the use of related therapies, such as corticosteroids, which are commonly used to control the side effects of chemotherapy, as they have been found to reduce TLS density in lung squamous cell carcinoma and impair positive clinical impact (160). In another report, the presence of tumour associated TLSs was initially associated with a favourable response to neoadjuvant chemotherapy in breast cancer patients (161). Similarly, the density of tumour associated TLSs in HER2+ breast cancer was strongly associated with DFS and responsiveness to adjuvant trastuzumab therapy (162). A study of 264 high-grade serous ovarian cancer (HGSC) patients from two cohorts and 340 HGSC cases from The Cancer Genome Atlas showed that CXCL13 plays a key role in shaping anti-TME by promoting the maintenance of CXCR5+CD8+ T cells in TLSs which supporting the idea that combination of CXCL13 and PD-1 in HGSC clinical study (163).

Tumour-infiltrating B cells are well characterized, but their overall functional role in cancer is not fully understood. Some studies suggest that they have a tumor-promoting role, while others suggest that they are positively associated with better cancer prognosis, especially when they are associated with organized lymphocyte aggregates (known as TLSs).

B cells and TLSs are potential biomarkers and therapeutic targets in response to ICB in patients with melanoma and renal cell carcinoma (136, 146). In a consistent manner, the existence of B cells in TLSs was related to improved survival and a high response rate to PD-1 blockade in soft tissue sarcoma patients (135). However, as the functional status of TLSs varies, the main contributors are to the induction of favourable TLSs that augment antitumour efficacy remain unknown.

A former neoadjuvant ICB trial in melanoma patients showed an enrichment of B-cell markers in tumours of patients who responded to treatment by targeted expression profiling compared to those in nonresponding patients (164). Immune checkpoint treatment of murine tumours increases the number and size of TA-TLSs and promotes classical organization in association with diminished tumour outgrowth (165).

In patients with metastatic melanomas, the co-existence of tumour-associated CD20+ B cells and CD8+ T cells was correlated with improved survival, which was revealed by immunofluorescence staining that the development of tertiary lymphoid structures was found in CD8+CD20+ tumours. Moreover, B cell-rich tumours were associated with increased levels of TCF7+ naive and memory T cells, suggesting that TLs showed a critical role in the immune microenvironment of melanoma, by imparting a distinct T cell phenotype (146). These observations suggested that TA-TLSs are important predictors of patient responses to chemo- and immunotherapies, along with the overall intratumoural CD8+ TILs, mutational burden, and PD-L1 expression (166). Whether this is a consequence of additional regulatory mechanisms and whether these operate within TA-TLSs remain to be determined.

## Problems and Dilemmas Regarding Hyperprogression

### Definition of and Diagnostic Criteria for HPD

ICI therapies consist not only of monoclonal antibodies targeting the traditional check point pathways (167), but they also include TIM3 antibodies (168) and B and T lymphocyte attenuator (BTLA) antibodies (169). However, ICIs were found to induce novel tumour responses, such as hyperprogression and pseudoprogression.

The occurrence of HPD after ICI treatment was initially characterized in 2016 (170). Then a number of cases of HPD after ICI treatment have been described. The novel pattern of tumour response is a potentially harmful side effect of checkpoint blockade therapy that can accelerate disease progression in a subset of patients (171). In contrast to HPD, pseudoprogression may indicate a good treatment effect. HPD can be defined as primary drug resistance with a high incidence, ranging from 4% to 29% according to the different algorithmic approaches and tumour types used (172). However, the mechanism of actions (MOAs) of hyperprogression remain largely unknown.

It has been suggested that amplification of MDM2/4 gene, EGFR gene mutation and chromosome 11 region 13 (CCND1/FGF3/FGF4/FGF19) may be associated with the development of HPD. When overexpression of the ubiquitin ligase MDM2 can disrupt its regulation of wild-type (WT) p53, it blocks the activation of the transcriptional domain of the p53 gene and leads to p53 inactivation through down-regulating of the ubiquitin-dependent p53 proteins (11). In addition, PD-1 and PD-L1 inhibitors can induce upregulation of IFN-γ and activation of the JAK-STAT signalling pathway, leading to the expression of IFN regulatory factor 8 (IRF8). Anchoring of IRF8 to the MDM2 promoter mediates its expression, which may also lead to HPD (11, 173). From a clinical point of view, tumour growth rate (TGR), tumour growth kinetics (TGKR) and time to treatment failure (TTF) were used as valid algorithmic methods to define high progression and all the different methods are summarized in **Table 2**.

**Table 2:** Different criteria for HPD from clinical perspective [adapted from Table from the paper of Hongjing et al. (174)].

Name	Cancer types	Applications	Criteria	Advantages	Disadvantages	Reference
TGR_R_	Solid tumours	PD-1/PD-L1 inhibitors	TGR_R_ ≥2	First HPD definition	Pre-ICI treatments details are needed	(175)
TGK_R_	R/M HNSCC	PD-1/PD-L1 inhibitors	TGK_R_ ≥2	Pseudoprogression and HPD can be distinguished	Pre-ICI treatments details are needed	(176)
Kato et al. criteria	Multiple types of solid tumours	Immunotherapy agents	TTF < 2 months; 50% increase in tumour burden; >2-fold change in progression rate	Need less time for HPD characteristics	Clinical status changes are ignored	(11)
Lo Russo et al. criteria	Multiple types of solid tumours	ICIs	TTF < 2 months; 50% increase in tumour lesions; ≥ 2 new lesions; spread of disease; clinical deterioration by ECOG	Applicable for first-line treatment with ICIs	Higher false positive rate	(12)

The pathophysiological MOA of HPD is still unknown to a large extent. Nevertheless, an accumulating number of investigations suggest that alterations in the TME during checkpoint therapy, for example, activation of PD-1-expressing Treg cells and CD8+ T cells, may initiate an increase in accelerated tumour development. In addition, changes in the tumour immune microenvironment, aggravation of innate immunosuppression, activation of carcinogenic signals, and regulation of tumour-promoting cytokines may be critical for the development of HPD (177).

More recently, Champiat et al. (175) proposed several hypotheses for the development of HPD during immunotherapy. For example, a) blockade of immune checkpoints- has the possibility to stimulate -Tregs functionally, locally forming an immunosuppressive TME, i.e., enhanced reparations of negative regulatory signals further aggravate T cell exclusion. b) blockade of immune checkpoints induces polarization of immunosuppressive cells, such as M2 macrophages, dendritic cells or bone marrow cells, producing large amounts of immunosuppressive cytokines; c) blockade of immune checkpoints leads to stimulation of Th1 and Th17-mediated inflammatory reactions or activation of specific oncogenic pathways, thereby establishing conditions for faster tumour development and resistance to immunotherapy.

### Potential Predictors and Biomarkers of HPD

As tumour mutations and other genetic tests are widely used as potential biomarkers in immunotherapy, related technologies are also used in the prediction of HPD. For instance, Kato et al. performed NGS on a variety of tumour types from 155 patients. MDM2/MDM4 amplifications were found in six patients which had a TTF of <2 months, and 4/6 experienced hyperprogression. EGFR alterations were observed in ten patients; eight had a TTF of <2 months, and two experienced hyperprogression (11). The same authors also published a separate report indicated that in a patient with gastroesophageal junction adenocarcinoma MDM2 and EGFR amplifications was found, 3.5% out of 100,000 samples had MDM2 amplification (10). Another study of four patients with hyperprogression revealed MDM2/MDM4 amplifications in two patients and EGFR amplification in one patient (178). In contrast, Kim et al. found no MDM2/MDM42 amplifications in the 18 patients with hyperprogression, also no significant differences in the EGFR amplification rates were found; but interestingly, three other genes STK11 (28 *vs* 3%), JAK3 (22 *vs* 2%) and SOX9 (17 *vs* 1%) to be more frequent in patients with hyperprogression than in those without hyperprogression (179). No STK11 to be associated with a TTF <2 months was found by Kato et al. (11).

No additional studies on these genes were reported, and more work is necessary to clarify the roles of various genetic mutations in hyperprogression. Liquid biopsies that detect cell-free DNA (cfDNA) or circulating tumour DNA (ctDNA) show promise as potential biomarkers for immunotherapy (180), for example, the recent trend of MRD technology Chromosomal instability has been linked to poor prognosis and treatment resistance in several malignancies (181).

Although MDM2 amplification and TP53 mutations have been shown to indicate HPD by some researchers, other studies have shown that advanced gastric tumour patients without HPD also exhibit genetic changes, such as ERBB2 amplification, MDM2 amplification, TP53 mutations, KRAS amplification, and PIK3CA mutations, indicating that these changes may not be HPD-specific (182). The emergence of the above controversial genomic results suggests the need for larger cohort studies or retrospective studies for the prediction of HPD gene levels (182). In addition, changes in cellular levels in tumour patients may also be a factor affecting HPD. Zuazo-Ibarra et al. examined highly differentiated CD28- CD27- CD4 T (THD) cells using FACS in the peripheral blood of 34 patients with NSCLC both prior to starting and during PD-1/PD-L1 inhibitor treatment. A low percentage of THD cells at baseline was found in 70% (19 of 27) of patients with no objective response and in 0% patients with an objective response (p = 0.008). A low percentage of THD cells at baseline was present in 100% (7 of 7) of hyperprogression patients *versus* 43% (6 of 14) of standard progression patients (p = 0.01) (183). Meanwhile, Kim et al. focused on peripheral blood CD8+ T lymphocytes to identify potential predictors and indicated that the number of effector or memory CD8+ T lymphocytes (CCR7−CD45RA−) was reduced (184, 185), while exhausted tumour-reactive CD8+ T lymphocytes (TIGIT+ PD-1+) reached to a high level in hyperprogressive NSCLC patients (186). In addition, both biomarkers can be used as independently predict clinical results based on PFS and OS. The above data suggest that the level of pre-existing antitumour resistance immunity and the severe degree of T-cell depletion can be used as predictive indicators of HPD.

## Summary

Although ICIs have substantially progressed the treatment of cancer in recent years, tumour progression due to immune resistance remains a substantial challenge for oncology treatment. Several issues deserve deeper consideration, such as the TME in immunotherapy-resistant cancers having multiple immunosuppressive signals that must be bypassed to achieve a clinical response; a better understanding of the heterogeneity within tumours from the same patient; and the requirement of high-quality T cell induction for immune checkpoint function. Additionally, the interaction of draining lymph nodes with TLSs during tumour progression and immune cell infiltration suggest that the subtle structure of the TME may be equally important, not only for the T cells activation but also the impacts of CAFs and macrophages are important to build up the whole TME. Previous research has focused on tumour and immune cell suppression mechanisms within the tumour, but it is increasingly recognized that tumour and immune suppressor cells interact with stromal cells to form a complex signalling network that may also be essential for T cell exclusion. An increasing number of studies have also elucidated the roles of stromal cells in promoting immune evasion and supporting cancer progression and metastasis, the introduction of the concept of spatial-omics has also enhanced the understanding of researchers in this field (**Figure 1**).

In the search for a cancer cure, ICIs are potentially the best treatment developed in recent years but may not be the final end point. The evolutionary process of tumours serving as a microenvironment for development has become more complex over time. Current immunotherapies rely on a snapshot of tumours at a certain time point as a cut-off, which is why some chemotherapeutic and ICI treatment strategies must be continually altered. It is believed that a better understanding of tumours at the molecular, protein and cellular levels as well as over time will lead to more appropriate treatments. This is why precision medicine and companion diagnoses are particularly important.

## Author Contributions

SW contributed to the idea generation and article writing. KX contributed to the article writing and wording. TL contributed to the overall design, idea generation and article writing. SW and TL contributed equally to this work. All authors contributed to the article and approved the submitted version.

## Funding

This paper was supported by the Foundation of Scientific Research Project of Education Department of Jilin Province (JJKH20201177KJ, China) and the Foundation of Department of Human Resources and Social Security of Jilin Province (2020009, China). We also thank the China Scholarship Council for supporting the overseas research program of KX (No.201508130060) and TL (No.201308130088).

## Conflict of Interest

The authors declare that the research was conducted in the absence of any commercial or financial relationships that could be construed as a potential conflict of interest.

## Glossary

**Table:**

ICI	Immune checkpoint inhibitor
TME	Tumour microenvironment
CPI	Cancer checkpoint inhibitor
PD-1	Programmed cell death 1
PD-L1	PD-1 ligand 1
TMB	Tumour mutational burden
TLS	Tertiary lymphoid structures
*ORR*	Objective response rate
IFN-γ	Interferon-γ
*CTLA-4*	Cytotoxic T lymphocyte associated antigen-4
*TIL*	Tumor infiltrating lymphocyte
HPD	Hyperprogressive diseases
MDM	Murine double minute
*CCND1*	Cyclin D1
*FGF*	Fibroblast growth factor
EGFR	Pidermal growth factor receptor
WES	Whole-exome sequencing
WGS	Whole-genome sequencing
TSA	Tumour-specific antigen
TAA	Tumour-associated antigen
NSCLC	Non-small-cell lung cancer
OS	Overall survival
PFS	Progression-free survival
MSI-H	High microsatellite instability
dMMR	Deficient DNA mismatch repair
CRC	Colorectal cancer
RCC	Renal cell cancer
MCC	Merkel cell carcinoma
TCR	T cell receptor
β2M	Beta 2 microglobulin
HLA	Human leukocyte antigen
LMP	Large multifunctional protease
TAP	Transporter-associated with antigen processing
MSS	Microsatellite stable
MHC	Histocompatibility complex
*TGF-β*	Transforming growth factor-β
CAF	Cancer-associated fibroblast
*SNAI1*	Snail Homolog 1
*HLA*	Human leukocyte antigen
LOH	HLA loss of heterozygosity
ITH	Intratumour heterogeneity
HPV	Human papillomavirus
EBV	Epstein-Barr virus
HTLV 1	Human T cell leukaemia virus
HL	Hodgkin lymphoma
NHL	Non-Hodgkin lymphoma
NPC	Nasopharyngeal carcinoma
LMP	Latent membrane protein
*EBNA1*	Ebv-determined nuclear antigen 1
VST	Virus-specific T cell
NPC	Nasopharyngeal carcinoma
CTA	Cancer/testis antigen
CTL	Cytotoxic T lymphocyte
ITH	Increased tumour heterogeneity
NGS	Next-Generation Sequencing
ECM	Extracellular matrix
IPRES	Innate anti-PD-1 resistance
*MAPK*	Mitogen-activated protein kinase
DDR	DNA damage repair
ATM	Ataxia telangiectasia mutated
*N*K cell	Natural killer *cell*
Tregs	T regulatory cells
MDSC	Myeloid-derived suppressor cell
TAM	Tumour-associated macrophage
*CXCL*	C-X-C chemokine ligand
CCL	Chemoattractant cytokine ligand
OSCC	Oral squamous cell carcinoma
MCP-1	Macrophage chemoattractant protein-1
GM-CSF	Granulocyte-macrophage stimulating factor
HNSCC	Head and neck squamous cell carcinoma
*HIF-1*α	Hypoxia-inducible factor-1α
CAFs	Cancer-associated fibroblasts
cAMP	Cyclic adenosine monophosphate
PRRs	Pattern recognition receptors
FMT	Fecal microbiota transplant
APCs	Antigen presenting cells
HEVs	High endothelial venules
STAT1	Signal transducer and activator of transcription 1
IRF7	Interferon regulatory factor 7
CX3CL1	C-X3-C motif chemokine ligand 1
DCs	Dendritic cells
SLOs	Secondary lymphoid organs
BALT	Bronchial associated lymphoid tissue
DFS	Disease-free survival
HGSC	High-grade serous ovarian cancer
ICB	Immune checkpoint blockade
BTLA	B and T lymphocyte attenuator
TCF7	Transcription factor 7
TIM3	T cell immunoglobulin mucin 3
MOAs	Mechanism of Actions
IRF8	IFN regulatory factor 8
TGR	Tumour growth rate
TGK	Tumour growth kinetics
TTF	Time to treatment failure
cfDNA	Cell-free DNA
ctDNA	Circulating tumour DNA
